# Health in the Philippines

**Published:** 1907-04

**Authors:** 


### HEALTH IN THE PHILIPPINES.

The comparative freedom from disease which residents of the Philippine Islands who are willing to follow the few simple rules recommended by the Bureau of Health have enjoyed during the year, although constantly exposed to highly communicable diseases, the mortality of which is exceedingly great, is another most convincing proof of the value of health organizations when health legislation places them in position to carry out modern scientific sanitary methods. In other words, for an expenditure of 5 centavos (2 1-2 cents, United States currency) per capital per annum, based upon a population of 6,500,000, over 1,000,000 persons have been protected against smallpox, and of over 1,600,000 living in the provinces exposed to cholera, only 4,143 contracted the disease, which means that only dozens were seized where hundreds were attacked before.

Quinine was distributed free to persons living in malarious districts and good health was enjoyed by thousands where sickness and death were ever present in the past. Many water courses from which town water supplies are obtained were protected from pollution, thus adding many more thousands to those enjoying good health instead of being afflicted with water-borne diseases. Vigorous measures were enforced to enable the public to obtain good meat. The few cases of measles and diphtheria which came to these shores were carefully isolated to prevent the spread of the infection, and thus the Philippines were protected from at least two diseases which have not as yet gained a foothold. In order to prevent that dreadful scourge of modern civilization—typhoid fever—from establishing itself in the Philippine Islands, all cases of this disease were searched for and measures taken to disinfect discharges from such patients in order that there might be no spread; and in many, many other ways measures were taken which prevented persons from contracting disease, and thus the work of the Bureau of Health has added very much to the sum total of happiness which was enjoyed by the people of these Islands, as well as contributing to the economic wealth of the country by increasing the producing power of the people.

Each succeeding year of experience in health work shows very clearly that the white man's chances of contracting disease in the Philippine Islands are less than they are in the United States. During the year the death rate among Americans was only 9.34 per thousand, and even if several per cent. is added for those who left here sick and subsequently died in the United States, the death rate will still not be higher than in the most salubrious communities in the United States.

To keep the cholera from becoming epidemic, and to confine it to as small a territory as possible, has required the best efforts of the officers and employees of the Bureau of Health during the year. In almost every instance where it was impossible to institute measures against its suppression, the disease assumed threatening proportions, and it was only with the greatest difficulty in those instances that it was brought under control. While it is satisfactory to report that cholera was confined to narrower limits, and less persons affected thereby than has been the case previously in the Philippine Islands, yet is is very discouraging for a health officer to be compelled to devote the greater portion of his efforts to keeping a disease in check, which, even if succcessful, does nothing to improve the health conditions in the Islands in a permanent way. In other words, if the energy which has been expended in combating cholera could have been devoted during the year to making permanent sanitary improvements which would make such diseases as cholera impossible, a long step in advance would have been taken instead of our energy being dissipated and nothing permanent being accomplished. Yet, in spite of the fact that so much time was required in combating cholera, more work was eccamplished than in any year since the Bureau of Health has been established, more persons vaccinated, more sick were treated, more permanent sanitary improvements were made, more medicine was distributed, more nuisances abated, more free dispensaries were operated, and less plague, less smallpox, and less beriberi in public institutions occurred than has heretofore been the case in an yone year, and, what is also well worth mentioning, is the fact that these results were obtained with smaller appropriations than have been expended yearly during the preceding four years.—An. Report, Bureau of Health for the Philippines, 1906.

